# Synthesis and crystal structure of *N*-phenyl-2-(phenyl­sulfan­yl)acetamide

**DOI:** 10.1107/S2056989024002573

**Published:** 2024-03-26

**Authors:** Reham A. Mohamed-Ezzat, Benson M. Kariuki, Galal H. Elgemeie

**Affiliations:** a Chemistry of Natural & Microbial Products Department, Pharmaceutical and Drug Industries Research Institute, National Research Center, Cairo, Egypt; b School of Chemistry, Cardiff University, Main Building, Park Place, Cardiff CF10, 3AT, United Kingdom; c Department of Chemistry, Faculty of Science, Helwan University, Helwan, Cairo, Egypt; Universität Greifswald, Germany

**Keywords:** crystal structure, acetamide, sulfide, synthesis

## Abstract

*N*-Phenyl-2-(phenyl­sulfan­yl)acetamide, C_14_H_13_NOS, was synthesized and structurally characterized. In the crystal, N—H⋯O hydrogen bonding leads to the formation of chains of mol­ecules along the [100] direction. The chains are linked by C—H⋯π inter­actions, forming a three-dimensional network. The crystal studied was twinned by a twofold rotation around [100].

## Abstract 2

In the crystal of the title compound, N—H⋯O hydrogen bonds form chains of mol­ecules along the [100] direction. The chains are linked by C—H⋯π inter­actions, forming a three-dimensional network.

## 1. Chemical context

The acetamide moiety possesses therapeutic potential for targeting various diseases. Acetamide-containing drugs are used for inflammation control, cyclo­oxygenase (COX) enzyme inhibition, and as anti­viral drugs (Agrawal *et al.*, 2010 ▸; Orzalesi *et al.*, 1977 ▸). Recently, starting from acetamides, we have synthesized various heterocyclic compounds that exhibit diverse activities, including anti-SARS CoV-2 (Mohamed-Ezzat & Elgemeie, 2023 ▸), anti­microbial (Elgemeie *et al.*, 2017*a* ▸,*b* ▸), anti­tumor properties (Elgemeie & Mohamed-Ezzat, 2022 ▸; Mohamed-Ezzat *et al.*, 2023*a* ▸,*b* ▸), as well as potential for other applications (Elgemeie *et al.*, 2015 ▸, 2017*a* ▸,*b* ▸, 2019 ▸; Mohamed-Ezzat *et al.*, 2021 ▸, 2023*a* ▸,*b* ▸).

Additionally, the evolution of the pharmaceutical industry has been greatly aided by the discovery of sulfur-based therapies. Sulfur-derived functional groups can be found in a broad range of natural products and pharmaceuticals. Sulfur remains the dominant heteroatom integrated into a variety of FDA-approved sulfur-containing medications (Feng *et al.*, 2016 ▸).

Sulfides have been presented *inter alia* as precursors for sulfonyl chloride synthesis (Langler *et al.*, 1979 ▸). Advanced methods previously reported for the transformation of sulfides include, for example, using sulfate-modified multi-walled carbon nanotubes (S-MWCNT) and mesoporous carbon (S-MC) as heterogenous catalysts to facilitate the synthesis of acetamide derivatives (Minchitha *et al.*, 2018 ▸). 
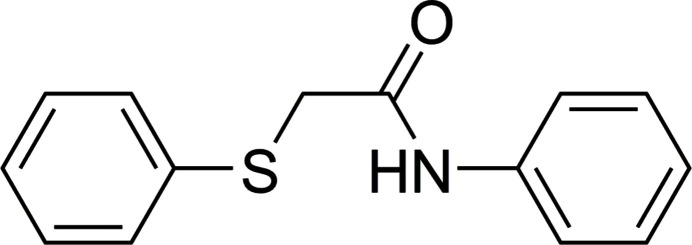


Herein, we report the first synthesis of a sulfide from a sulfonyl derivative *via* an alternative new, direct and efficient approach. Upon reaction of the sulfonyl­guanidine derivative with 2-chloro-*N*-phenyl­acetamide, the title compound *N*-phenyl-2-(phenyl­sulfan­yl)acetamide (**3**) is formed. Its chemical structure was confirmed by spectroscopic techniques and elemental analysis. The ^1^H NMR spectrum has a singlet signal of the methyl­ene group at δ 3.84 ppm, the multiplet aromatic protons at δ 7.30 ppm, as well as the amine proton at δ 9.15 ppm, which is roughly in accordance with previously reported data (Motherwell *et al.*, 2002 ▸). Confirmation of the mol­ecular structure is provided by means of single crystal X-ray diffraction structural analysis which provides the first crystal structure and geometric parameters for the title compound.

## 2. Structural commentary

**Figure 1:**
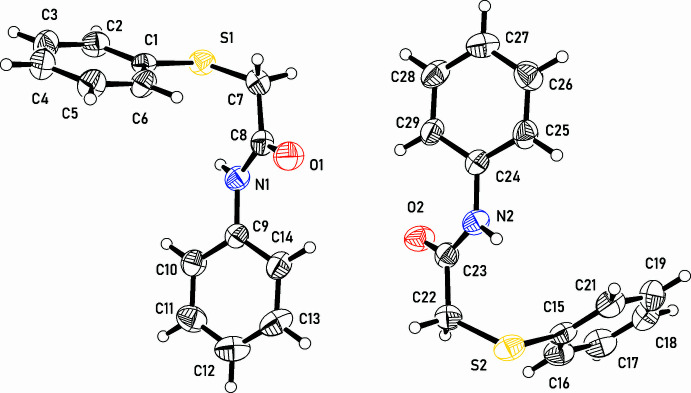
The asymmetric unit and mol­ecular structures of the two independent mol­ecules of *N*-phenyl-2-(phenyl­sulfan­yl)acetamide (**3**) showing displacement ellipsoids at the 50% probability level.

The asymmetric unit of the crystal structure is composed of two independent mol­ecules of the title compound (Fig. 1 ▸). The mol­ecules of **3** consist of three planar segments, namely sulfanyl­benzene [**sb_1_** (C1–C6/S1) and **sb_2_** (C15–C21/S2)], acetamide [**ac_1_** (C7/C8/N1/O1) and **ac_2_** (C22/C23/N2/O2)], and phenyl [**ph_1_** (C9–C14) and **ph_2_** (C24–C29)] groups. The conformations of the two independent mol­ecules in the structure are similar but not identical. The twist angles **sb**/**ac** are 85.12 (11) and 77.58 (11)° for mol­ecules **1** and **2**, respectively, and twist angles **sb**/**ph** are 28.30 (10) and 30.60 (10)° for mol­ecules **1** and **2**, respectively. Thus, the phenyl and acetamide groups are almost coplanar whereas the sulfanyl­benzene groups are almost perpendicular to this plane. The C_phen­yl_—S-C—C_carbon­yl_ torsion angles are 72.1 (3)° for C1—S1—C7—C8 and −65.13 (3)° for C15—S2—C22—C23. A similar mol­ecular conformation is observed in the crystal structures of the related compounds *N*-(2-hy­droxy-5-chloro­phen­yl)thio­phenyl­acetamide (Tarimci *et al.*, 1998 ▸) and 2-[(2-amino­phen­yl)sulfan­yl]-*N*-(2-nitro­phen­yl)acetamide (Murtaza *et al.*, 2019 ▸) in which the C_phen­yl_—S—C—C_carbon­yl_ torsion angles are *ca* 80°.

## 3. Supra­molecular features

**Figure 2:**
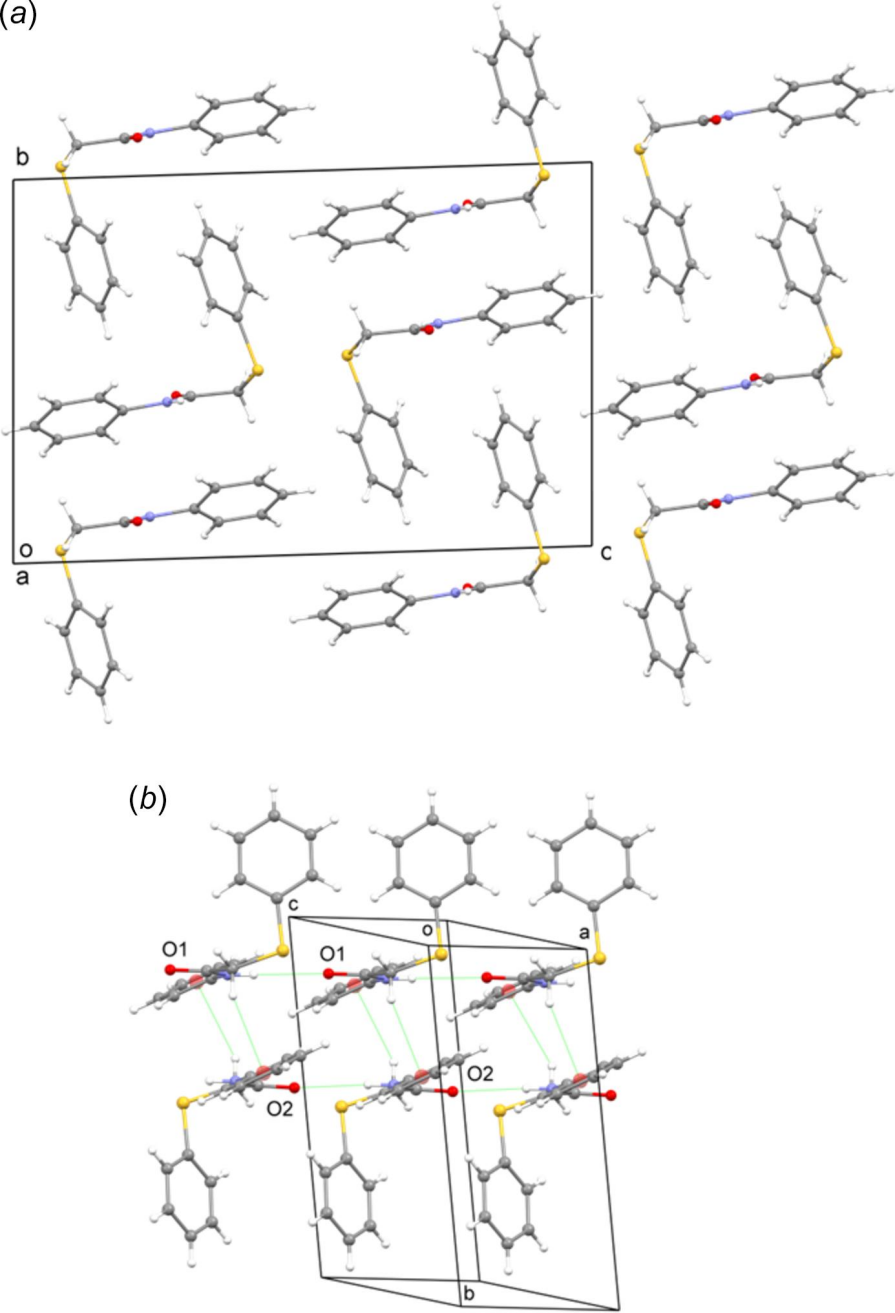
(*a*) Crystal packing in the crystal structure of *N*-phenyl-2-(phenyl­sulfan­yl)acetamide (**3**). (*b*) A segment of the crystal structure of compound **3** showing the N—H⋯O and C—H⋯π inter­molecular contacts as green dotted lines.

The packing in the crystal structure of **3** is shown in Fig. 2 ▸ *a*. In the crystal, the acetamide groups of each set of independent mol­ecules inter­act through weak N—H⋯O contacts (Table 1 ▸), forming chains parallel to [100] (Fig. 2 ▸ *b*).

Adjacent chains are linked by weak C—H⋯π contacts between methyl­ene and phenyl groups. The rings involved in the contacts are **ph_1_** (C9–C14, *Cg*1) and **ph_2_** ^#^ (C24–C29, *Cg*2^#^) where # is *x* + 1, *y*, *z*. The associated H⋯π distances H7*A*⋯**ph_2_** ^#^, and H22*B*⋯**ph_1_** are 2.80 Å and 2.94 Å, respectively. The H⋯centroid distances H7*A*⋯*Cg*2^#^ and H22*B*⋯*Cg*1 are 3.00 and 3.10 Å, respectively. The C—H⋯centroid angles for C7—H7*A*⋯*Cg*2^#^ and C22—H22*B*⋯*Cg*1 are 129 and 128°, respectively.

## 4. Database survey

A search of the CSD (version 5.44, April 2023; Groom *et al.*, 2016 ▸) using the routine ConQuest (Bruno *et al.*, 2002 ▸) for crystal structures containing the *N*-phenyl-2-(phenyl­sulfan­yl)acetamide fragment returned *N*-(2-hy­droxy-5-chloro­phen­yl)thio­phenyl­acetamide (NILWEK; Tarimci *et al.*, 1998 ▸) and 2-[(2-amino­phen­yl)sulfan­yl]-*N*-(2-nitro­phen­yl)acetamide (NULZOM; Murtaza *et al.*, 2019 ▸), which both have similar conformational geometries to compound **3**. In contrast, 2-[(2-amino­phen­yl)sulfan­yl]-*N*-(4-meth­oxy­phen­yl)acetamide (PAXTEP; Murtaza *et al.*, 2012 ▸) has a C_phen­yl_—S—C—C_carbon­yl_ torsion angle of 159° compared to the values of *ca* 80° in NILWEK and NULZOM and even more acute ones are observed in the crystal of the title compound.

## 5. Synthesis and crystallization

**Figure 3:**
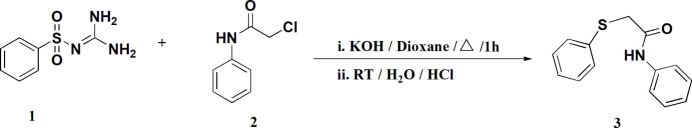
The synthesis of compound **3** from sulfonyl­guanidine.

A mixture of benzene­sulfonyl­guanidine (**1**) (0.01 mol) with 2-chloro-*N*-phenyl­acetamide **2** (0.01 mol) in dry 1,4-dioxane (20 mL) containing potassium hydroxide (0.015 mol) was refluxed for 1 h. The reaction mixture was poured onto ice–water and then neutralized using hydro­chloric acid (Fig. 3 ▸).

The solid precipitate that formed was then filtered, washed thoroughly with water and left in the open to dry at room temperature. The solid obtained was recrystallized from water to afford colorless crystals of compound **3** in 83% yield; mp > 573 K; ^1^H NMR (400 MHz, DMSO-*d_6_*): δ 3.84 (*s*, 2H, CH_2_), 7.30 (*m*, 10H, Ar-H), 9.15 (*s*, 1H, NH); analysis calculated for C_14_H_13_NOS (243.32): C, 69.11; H, 5.39; N, 5.76; S, 13.18. Found: C, 69.07; H, 5.35; N, 5.75; S, 13.16.

## 6. Refinement

Crystal data, data collection and structure refinement details are summarized in Table 2 ▸. The crystal studied was twinned by a twofold rotation around [100]. This problem was addressed using a HKLF5 file for refinement. The N-bound hydrogen atoms were refined with regard to location while the displacement parameters were constrained to those of their parent atoms [*U*_iso_(H) = 1.2*U*_eq_(N)]. All other hydrogen atoms were placed in idealized positions (C—H = 0.93–0.97 Å) and refined using a riding model with *U*_iso_(H) = 1.2*U*_eq_(C).

## Supplementary Material

Crystal structure: contains datablock(s) I. DOI: 10.1107/S2056989024002573/yz2049sup1.cif

Structure factors: contains datablock(s) I. DOI: 10.1107/S2056989024002573/yz2049Isup2.hkl

Supporting information file. DOI: 10.1107/S2056989024002573/yz2049Isup3.cml

CCDC reference: 2311395

Additional supporting information:  crystallographic information; 3D view; checkCIF report

## Figures and Tables

**Table 1:** Hydrogen-bond geometry (Å, °)

*D*—H⋯*A*	*D*—H	H⋯*A*	*D*⋯*A*	*D*—H⋯*A*
N1—H1⋯O1^i^	0.86 (3)	2.70 (3)	3.477 (3)	152 (3)
N2—H2*A*⋯O2^ii^	0.83 (3)	2.71 (4)	3.456 (3)	150 (3)

Symmetry codes: (i) 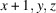 ; (ii) 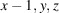 .

**Table 2:** Experimental details

Crystal data	
Chemical formula	C_14_H_13_NOS
*M*_r_	243.31
Crystal system, space group	Triclinic, *P* 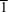
Temperature (K)	293
*a*, *b*, *c* (Å)	5.6768 (3), 12.0747 (6), 18.1912 (9)
α, β, γ (°)	87.071 (4), 82.110 (4), 81.110 (4)
*V* (Å^3^)	1219.72 (11)
*Z*	4
Radiation type	Mo *K*α
μ (mm^−1^)	0.25
Crystal size (mm)	0.54 × 0.17 × 0.09

Data collection	
Diffractometer	Agilent SuperNova, Dual, Cu at home/near, Atlas
Absorption correction	Multi-scan (*CrysAlis PRO*; Rigaku OD, 2023 ▸)
*T*_min_, *T*_max_	0.662, 1.000
No. of measured, independent and observed [*I* > 2σ(*I*)] reflections	7742, 7742, 5708
*R*_int_	0.040
(sin θ/λ)_max_ (Å^−1^)	0.697

Refinement	
*R*[*F* ^2^ > 2σ(*F* ^2^)], *wR*(*F* ^2^), *S*	0.058, 0.162, 1.03
No. of reflections	7742
No. of parameters	314
H-atom treatment	H atoms treated by a mixture of independent and constrained refinement
Δρ_max_, Δρ_min_ (e Å^−3^)	0.23, −0.23

Computer programs: *CrysAlis PRO* (Rigaku OD, 2023 ▸), *SHELXT* (Sheldrick, 2015*a* ▸), *SHELXL* (Sheldrick, 2015*b* ▸) and *ORTEP-3 for Windows* and *WinGX* (Farrugia, 2012 ▸).

## supplementary crystallographic information

### Crystal data

**Table:**

C_14_H_13_NOS	*Z* = 4
*M**_r_* = 243.31	*F*(000) = 512
Triclinic, *P*1	*D*_x_ = 1.325 Mg m^−^^3^
*a* = 5.6768 (3) Å	Mo *K*α radiation, λ = 0.71073 Å
*b* = 12.0747 (6) Å	Cell parameters from 3492 reflections
*c* = 18.1912 (9) Å	θ = 3.7–28.1°
α = 87.071 (4)°	µ = 0.25 mm^−^^1^
β = 82.110 (4)°	*T* = 293 K
γ = 81.110 (4)°	Needle, yellow
*V* = 1219.72 (11) Å^3^	0.54 × 0.17 × 0.09 mm

### Data collection

**Table:**

Agilent SuperNova, Dual, Cu at home/near, Atlas diffractometer	5708 reflections with *I* > 2σ(*I*)
ω scans	*R*_int_ = 0.040
Absorption correction: multi-scan (CrysAlisPro; Rigaku OD, 2023)	θ_max_ = 29.7°, θ_min_ = 3.4°
*T*_min_ = 0.662, *T*_max_ = 1.000	*h* = −7→6
7742 measured reflections	*k* = −15→15
7742 independent reflections	*l* = −24→24

### Refinement

**Table:**

Refinement on *F*^2^	Primary atom site location: structure-invariant direct methods
Least-squares matrix: full	Hydrogen site location: mixed
*R*[*F*^2^ > 2σ(*F*^2^)] = 0.058	H atoms treated by a mixture of independent and constrained refinement
*wR*(*F*^2^) = 0.162	*w* = 1/[σ^2^(*F*_o_^2^) + (0.0737*P*)^2^ + 0.551*P*] where *P* = (*F*_o_^2^ + 2*F*_c_^2^)/3
*S* = 1.03	(Δ/σ)_max_ = 0.001
7742 reflections	Δρ_max_ = 0.23 e Å^−^^3^
314 parameters	Δρ_min_ = −0.23 e Å^−^^3^
0 restraints	

### Special details

**Table:**

Experimental. Single-crystal XRD data were collected at room temperature on an Agilent SuperNova Dual Atlas diffractometer using mirror-monochromated Mo Kα radiation.
Geometry. All esds (except the esd in the dihedral angle between two l.s. planes) are estimated using the full covariance matrix. The cell esds are taken into account individually in the estimation of esds in distances, angles and torsion angles; correlations between esds in cell parameters are only used when they are defined by crystal symmetry. An approximate (isotropic) treatment of cell esds is used for estimating esds involving l.s. planes.
Refinement. Refined as a 2-component twin.

### Fractional atomic coordinates and isotropic or equivalent isotropic displacement parameters (Å^2^)

**Table:**

	*x*	*y*	*z*	*U*_iso_*/*U*_eq_	
C1	1.1641 (5)	−0.1130 (2)	0.11013 (13)	0.0415 (6)	
C2	1.3815 (5)	−0.1806 (3)	0.08899 (15)	0.0532 (7)	
H2	1.506625	−0.150022	0.060835	0.064*	
C3	1.4122 (6)	−0.2916 (3)	0.10934 (17)	0.0600 (8)	
H3	1.558973	−0.335965	0.095112	0.072*	
C4	1.2294 (6)	−0.3390 (3)	0.15062 (17)	0.0591 (8)	
H4	1.251613	−0.414900	0.164104	0.071*	
C5	1.0147 (6)	−0.2730 (3)	0.17151 (17)	0.0560 (8)	
H5	0.890629	−0.304415	0.199571	0.067*	
C6	0.9792 (5)	−0.1608 (2)	0.15170 (15)	0.0512 (7)	
H6	0.831850	−0.116989	0.166072	0.061*	
C7	0.8387 (6)	0.0859 (3)	0.10985 (15)	0.0551 (8)	
H7A	0.807561	0.158969	0.085390	0.066*	
H7B	0.741087	0.037878	0.090528	0.066*	
C8	0.7505 (6)	0.0994 (2)	0.19250 (15)	0.0480 (7)	
C9	0.8784 (5)	0.1283 (2)	0.31338 (14)	0.0434 (6)	
C10	1.0655 (5)	0.0928 (3)	0.35418 (16)	0.0534 (7)	
H10	1.212546	0.058503	0.330670	0.064*	
C11	1.0350 (7)	0.1082 (3)	0.42985 (17)	0.0648 (9)	
H11	1.161619	0.084293	0.457099	0.078*	
C12	0.8180 (7)	0.1586 (3)	0.46490 (18)	0.0689 (10)	
H12	0.796532	0.167685	0.515957	0.083*	
C13	0.6343 (6)	0.1953 (3)	0.42445 (18)	0.0658 (9)	
H13	0.488293	0.230248	0.448232	0.079*	
C14	0.6618 (6)	0.1812 (2)	0.34868 (17)	0.0557 (7)	
H14	0.535726	0.207188	0.321611	0.067*	
C15	0.6954 (5)	0.6251 (2)	0.38739 (14)	0.0483 (7)	
C16	0.8379 (6)	0.6999 (3)	0.40561 (17)	0.0594 (8)	
H16	0.958419	0.676480	0.435258	0.071*	
C17	0.8000 (7)	0.8093 (3)	0.37954 (19)	0.0677 (9)	
H17	0.897550	0.859209	0.391344	0.081*	
C18	0.6223 (6)	0.8462 (3)	0.33670 (19)	0.0655 (9)	
H18	0.598021	0.920754	0.320109	0.079*	
C19	0.4793 (6)	0.7723 (3)	0.31825 (19)	0.0667 (9)	
H19	0.357922	0.796749	0.289068	0.080*	
C21	0.5166 (6)	0.6616 (3)	0.34325 (17)	0.0579 (8)	
H21	0.421285	0.611422	0.330344	0.069*	
C22	1.0326 (6)	0.4309 (3)	0.38916 (15)	0.0568 (8)	
H22A	1.133364	0.478430	0.407477	0.068*	
H22B	1.070250	0.356802	0.411605	0.068*	
C23	1.1052 (6)	0.4220 (2)	0.30651 (15)	0.0467 (7)	
C24	0.9628 (5)	0.3906 (2)	0.18839 (14)	0.0405 (6)	
C25	0.7707 (5)	0.4253 (3)	0.14992 (16)	0.0529 (7)	
H25	0.626080	0.460022	0.174787	0.063*	
C26	0.7913 (6)	0.4091 (3)	0.07505 (18)	0.0619 (8)	
H26	0.661178	0.434012	0.049420	0.074*	
C27	1.0032 (6)	0.3561 (3)	0.03737 (16)	0.0604 (8)	
H27	1.017265	0.344880	−0.013342	0.072*	
C28	1.1923 (6)	0.3204 (3)	0.07640 (17)	0.0596 (8)	
H28	1.335639	0.284254	0.051660	0.071*	
C29	1.1747 (5)	0.3368 (2)	0.15161 (16)	0.0508 (7)	
H29	1.304784	0.311726	0.177232	0.061*	
N1	0.9169 (5)	0.1113 (2)	0.23607 (13)	0.0484 (6)	
H1	1.062 (6)	0.103 (3)	0.2146 (16)	0.058*	
N2	0.9320 (5)	0.4085 (2)	0.26590 (13)	0.0483 (6)	
H2A	0.790 (6)	0.419 (3)	0.2863 (17)	0.058*	
O1	0.5380 (4)	0.1003 (2)	0.21474 (12)	0.0656 (6)	
O2	1.3142 (4)	0.4238 (2)	0.28135 (11)	0.0618 (6)	
S1	1.14801 (15)	0.03015 (6)	0.08281 (4)	0.0539 (2)	
S2	0.72463 (16)	0.48404 (7)	0.42268 (4)	0.0584 (2)	

### Atomic displacement parameters (Å^2^)

**Table:**

	*U*^11^	*U*^22^	*U*^33^	*U*^12^	*U*^13^	*U*^23^
C1	0.0422 (15)	0.0485 (15)	0.0349 (12)	−0.0088 (13)	−0.0048 (11)	−0.0066 (11)
C2	0.0443 (17)	0.0652 (19)	0.0493 (15)	−0.0133 (15)	0.0039 (13)	−0.0050 (14)
C3	0.0466 (18)	0.061 (2)	0.0661 (18)	0.0059 (16)	0.0017 (15)	−0.0047 (15)
C4	0.057 (2)	0.0507 (17)	0.0667 (19)	−0.0023 (16)	−0.0053 (16)	−0.0001 (14)
C5	0.0492 (18)	0.0543 (18)	0.0622 (18)	−0.0114 (15)	0.0047 (15)	−0.0020 (14)
C6	0.0398 (16)	0.0535 (17)	0.0564 (16)	−0.0038 (14)	0.0059 (13)	−0.0061 (13)
C7	0.0588 (19)	0.0538 (17)	0.0507 (16)	−0.0015 (15)	−0.0099 (14)	0.0036 (13)
C8	0.0507 (18)	0.0408 (15)	0.0500 (15)	−0.0009 (14)	−0.0054 (14)	0.0007 (12)
C9	0.0444 (16)	0.0380 (14)	0.0481 (14)	−0.0100 (12)	−0.0025 (12)	−0.0018 (11)
C10	0.0424 (16)	0.0593 (18)	0.0573 (16)	−0.0067 (14)	−0.0027 (14)	−0.0027 (13)
C11	0.063 (2)	0.080 (2)	0.0559 (17)	−0.0175 (19)	−0.0163 (17)	−0.0002 (16)
C12	0.069 (2)	0.090 (3)	0.0513 (17)	−0.031 (2)	0.0012 (17)	−0.0101 (17)
C13	0.056 (2)	0.077 (2)	0.065 (2)	−0.0172 (18)	0.0091 (17)	−0.0246 (17)
C14	0.0471 (17)	0.0543 (18)	0.0642 (18)	−0.0019 (15)	−0.0043 (15)	−0.0125 (14)
C15	0.0422 (16)	0.0599 (17)	0.0417 (13)	−0.0109 (14)	0.0069 (12)	−0.0134 (12)
C16	0.058 (2)	0.070 (2)	0.0545 (17)	−0.0202 (17)	−0.0063 (15)	−0.0116 (15)
C17	0.073 (2)	0.065 (2)	0.070 (2)	−0.0277 (19)	−0.0017 (18)	−0.0116 (17)
C18	0.060 (2)	0.0559 (19)	0.074 (2)	−0.0052 (17)	0.0098 (18)	−0.0068 (16)
C19	0.0473 (19)	0.076 (2)	0.073 (2)	0.0004 (18)	−0.0070 (17)	−0.0035 (18)
C21	0.0455 (17)	0.067 (2)	0.0643 (18)	−0.0162 (16)	−0.0031 (15)	−0.0167 (16)
C22	0.0571 (19)	0.0625 (19)	0.0470 (16)	−0.0046 (16)	0.0001 (14)	0.0019 (14)
C23	0.0483 (17)	0.0410 (15)	0.0475 (15)	−0.0046 (13)	0.0011 (14)	0.0025 (12)
C24	0.0411 (15)	0.0329 (13)	0.0468 (14)	−0.0094 (12)	0.0013 (12)	−0.0017 (10)
C25	0.0381 (16)	0.0572 (18)	0.0602 (18)	−0.0038 (14)	0.0000 (14)	0.0000 (14)
C26	0.0539 (19)	0.073 (2)	0.0614 (19)	−0.0130 (17)	−0.0140 (16)	0.0021 (16)
C27	0.063 (2)	0.074 (2)	0.0471 (15)	−0.0232 (18)	0.0012 (15)	−0.0110 (14)
C28	0.0445 (18)	0.066 (2)	0.0653 (19)	−0.0062 (16)	0.0060 (15)	−0.0216 (15)
C29	0.0419 (16)	0.0496 (16)	0.0593 (17)	0.0012 (14)	−0.0058 (14)	−0.0126 (13)
N1	0.0421 (14)	0.0522 (14)	0.0491 (13)	−0.0056 (12)	−0.0002 (11)	−0.0045 (11)
N2	0.0395 (13)	0.0554 (14)	0.0475 (13)	−0.0088 (12)	0.0058 (11)	−0.0037 (10)
O1	0.0461 (13)	0.0878 (16)	0.0626 (13)	−0.0066 (12)	−0.0069 (11)	−0.0105 (11)
O2	0.0508 (13)	0.0819 (15)	0.0526 (11)	−0.0155 (12)	0.0021 (10)	−0.0084 (10)
S1	0.0569 (5)	0.0547 (4)	0.0488 (4)	−0.0124 (4)	0.0008 (4)	0.0004 (3)
S2	0.0579 (5)	0.0655 (5)	0.0487 (4)	−0.0162 (4)	0.0119 (4)	−0.0027 (3)

### Geometric parameters (Å, º)

**Table:**

C1—C2	1.388 (4)	C15—C21	1.385 (4)
C1—C6	1.389 (4)	C15—S2	1.781 (3)
C1—S1	1.767 (3)	C16—C17	1.375 (5)
C2—C3	1.363 (4)	C16—H16	0.9300
C2—H2	0.9300	C17—C18	1.366 (5)
C3—C4	1.376 (5)	C17—H17	0.9300
C3—H3	0.9300	C18—C19	1.378 (5)
C4—C5	1.368 (4)	C18—H18	0.9300
C4—H4	0.9300	C19—C21	1.385 (5)
C5—C6	1.375 (4)	C19—H19	0.9300
C5—H5	0.9300	C21—H21	0.9300
C6—H6	0.9300	C22—C23	1.508 (4)
C7—C8	1.527 (4)	C22—S2	1.800 (3)
C7—S1	1.791 (3)	C22—H22A	0.9700
C7—H7A	0.9700	C22—H22B	0.9700
C7—H7B	0.9700	C23—O2	1.215 (4)
C8—O1	1.217 (4)	C23—N2	1.342 (4)
C8—N1	1.342 (4)	C24—C25	1.376 (4)
C9—C10	1.380 (4)	C24—C29	1.378 (4)
C9—C14	1.384 (4)	C24—N2	1.420 (3)
C9—N1	1.414 (3)	C25—C26	1.373 (4)
C10—C11	1.382 (4)	C25—H25	0.9300
C10—H10	0.9300	C26—C27	1.381 (5)
C11—C12	1.373 (5)	C26—H26	0.9300
C11—H11	0.9300	C27—C28	1.370 (5)
C12—C13	1.364 (5)	C27—H27	0.9300
C12—H12	0.9300	C28—C29	1.380 (4)
C13—C14	1.382 (4)	C28—H28	0.9300
C13—H13	0.9300	C29—H29	0.9300
C14—H14	0.9300	N1—H1	0.86 (3)
C15—C16	1.382 (4)	N2—H2A	0.83 (3)
			
C2—C1—C6	118.9 (3)	C17—C16—H16	120.3
C2—C1—S1	116.2 (2)	C15—C16—H16	120.3
C6—C1—S1	124.9 (2)	C18—C17—C16	121.5 (3)
C3—C2—C1	120.2 (3)	C18—C17—H17	119.3
C3—C2—H2	119.9	C16—C17—H17	119.3
C1—C2—H2	119.9	C17—C18—C19	119.5 (3)
C2—C3—C4	121.0 (3)	C17—C18—H18	120.2
C2—C3—H3	119.5	C19—C18—H18	120.2
C4—C3—H3	119.5	C18—C19—C21	119.8 (3)
C5—C4—C3	119.0 (3)	C18—C19—H19	120.1
C5—C4—H4	120.5	C21—C19—H19	120.1
C3—C4—H4	120.5	C19—C21—C15	120.2 (3)
C4—C5—C6	121.1 (3)	C19—C21—H21	119.9
C4—C5—H5	119.5	C15—C21—H21	119.9
C6—C5—H5	119.5	C23—C22—S2	118.5 (2)
C5—C6—C1	119.8 (3)	C23—C22—H22A	107.7
C5—C6—H6	120.1	S2—C22—H22A	107.7
C1—C6—H6	120.1	C23—C22—H22B	107.7
C8—C7—S1	118.2 (2)	S2—C22—H22B	107.7
C8—C7—H7A	107.8	H22A—C22—H22B	107.1
S1—C7—H7A	107.8	O2—C23—N2	124.4 (3)
C8—C7—H7B	107.8	O2—C23—C22	118.9 (3)
S1—C7—H7B	107.8	N2—C23—C22	116.6 (3)
H7A—C7—H7B	107.1	C25—C24—C29	119.6 (3)
O1—C8—N1	124.2 (3)	C25—C24—N2	118.3 (2)
O1—C8—C7	119.3 (3)	C29—C24—N2	122.2 (3)
N1—C8—C7	116.5 (3)	C26—C25—C24	120.4 (3)
C10—C9—C14	119.4 (3)	C26—C25—H25	119.8
C10—C9—N1	118.5 (3)	C24—C25—H25	119.8
C14—C9—N1	122.0 (3)	C25—C26—C27	120.7 (3)
C9—C10—C11	120.2 (3)	C25—C26—H26	119.7
C9—C10—H10	119.9	C27—C26—H26	119.7
C11—C10—H10	119.9	C28—C27—C26	118.5 (3)
C12—C11—C10	120.1 (3)	C28—C27—H27	120.8
C12—C11—H11	119.9	C26—C27—H27	120.8
C10—C11—H11	119.9	C27—C28—C29	121.5 (3)
C13—C12—C11	119.7 (3)	C27—C28—H28	119.3
C13—C12—H12	120.2	C29—C28—H28	119.3
C11—C12—H12	120.2	C24—C29—C28	119.4 (3)
C12—C13—C14	121.0 (3)	C24—C29—H29	120.3
C12—C13—H13	119.5	C28—C29—H29	120.3
C14—C13—H13	119.5	C8—N1—C9	127.0 (3)
C13—C14—C9	119.5 (3)	C8—N1—H1	115 (2)
C13—C14—H14	120.2	C9—N1—H1	118 (2)
C9—C14—H14	120.2	C23—N2—C24	126.2 (2)
C16—C15—C21	119.5 (3)	C23—N2—H2A	118 (2)
C16—C15—S2	121.9 (2)	C24—N2—H2A	115 (2)
C21—C15—S2	118.5 (2)	C1—S1—C7	103.55 (14)
C17—C16—C15	119.4 (3)	C15—S2—C22	102.38 (14)
			
C6—C1—C2—C3	−0.5 (4)	S2—C15—C21—C19	176.3 (2)
S1—C1—C2—C3	178.3 (2)	S2—C22—C23—O2	155.3 (3)
C1—C2—C3—C4	0.4 (5)	S2—C22—C23—N2	−27.0 (4)
C2—C3—C4—C5	−0.3 (5)	C29—C24—C25—C26	−1.6 (4)
C3—C4—C5—C6	0.3 (5)	N2—C24—C25—C26	−179.5 (3)
C4—C5—C6—C1	−0.4 (5)	C24—C25—C26—C27	1.1 (5)
C2—C1—C6—C5	0.5 (4)	C25—C26—C27—C28	−0.1 (5)
S1—C1—C6—C5	−178.2 (2)	C26—C27—C28—C29	−0.3 (5)
S1—C7—C8—O1	−155.7 (3)	C25—C24—C29—C28	1.2 (4)
S1—C7—C8—N1	25.3 (4)	N2—C24—C29—C28	179.0 (3)
C14—C9—C10—C11	1.3 (4)	C27—C28—C29—C24	−0.2 (5)
N1—C9—C10—C11	179.9 (3)	O1—C8—N1—C9	−0.9 (5)
C9—C10—C11—C12	0.1 (5)	C7—C8—N1—C9	178.0 (2)
C10—C11—C12—C13	−1.2 (5)	C10—C9—N1—C8	153.0 (3)
C11—C12—C13—C14	0.8 (5)	C14—C9—N1—C8	−28.4 (4)
C12—C13—C14—C9	0.6 (5)	O2—C23—N2—C24	2.1 (5)
C10—C9—C14—C13	−1.6 (4)	C22—C23—N2—C24	−175.4 (2)
N1—C9—C14—C13	179.8 (3)	C25—C24—N2—C23	−152.4 (3)
C21—C15—C16—C17	−0.1 (4)	C29—C24—N2—C23	29.7 (4)
S2—C15—C16—C17	−177.0 (2)	C2—C1—S1—C7	173.7 (2)
C15—C16—C17—C18	0.8 (5)	C6—C1—S1—C7	−7.6 (3)
C16—C17—C18—C19	−0.8 (5)	C8—C7—S1—C1	72.1 (3)
C17—C18—C19—C21	0.0 (5)	C16—C15—S2—C22	−59.3 (3)
C18—C19—C21—C15	0.7 (5)	C21—C15—S2—C22	123.7 (2)
C16—C15—C21—C19	−0.7 (4)	C23—C22—S2—C15	−65.1 (3)

### Hydrogen-bond geometry (Å, º)

**Table:**

*D*—H···*A*	*D*—H	H···*A*	*D*···*A*	*D*—H···*A*
N1—H1···O1^i^	0.86 (3)	2.70 (3)	3.477 (3)	152 (3)
N2—H2*A*···O2^ii^	0.83 (3)	2.71 (4)	3.456 (3)	150 (3)

Symmetry codes: (i) *x*+1, *y*, *z*; (ii) *x*−1, *y*, *z*.
